# Integrated analysis of methylation-driven genes and pretreatment prognostic factors in patients with hepatocellular carcinoma

**DOI:** 10.1186/s12885-021-08314-5

**Published:** 2021-05-25

**Authors:** Dongsheng He, Shengyin Liao, Lifang Cai, Weiming Huang, Xuehua Xie, Mengxing You

**Affiliations:** Department of Medical Oncology, The First Hospital of Putian, Teaching Hospital, Fujian Medical University, Putian, 351100 China

**Keywords:** Hepatocellular carcinoma, Methylation-driven genes, Prognosis, Nomogram

## Abstract

**Background:**

The potential reversibility of aberrant DNA methylation indicates an opportunity for oncotherapy. This study aimed to integrate methylation-driven genes and pretreatment prognostic factors and then construct a new individual prognostic model in hepatocellular carcinoma (HCC) patients.

**Methods:**

The gene methylation, gene expression dataset and clinical information of HCC patients were downloaded from The Cancer Genome Atlas (TCGA) database. Methylation-driven genes were screened with a Pearson’s correlation coefficient less than − 0.3 and a *P* value less than 0.05. Univariable and multivariable Cox regression analyses were performed to construct a risk score model and identify independent prognostic factors from the clinical parameters of HCC patients. The least absolute shrinkage and selection operator (LASSO) technique was used to construct a nomogram that might act to predict an individual’s OS, and then C-index, ROC curve and calibration plot were used to test the practicability. The correlation between clinical parameters and core methylation-driven genes of HCC patients was explored with Student’s t-test.

**Results:**

In this study, 44 methylation-driven genes were discovered, and three prognostic signatures (LCAT, RPS6KA6, and C5orf58) were screened to construct a prognostic risk model of HCC patients. Five clinical factors, including T stage, risk score, cancer status, surgical method and new tumor events, were identified from 13 clinical parameters as pretreatment-independent prognostic factors. To avoid overfitting, LASSO analysis was used to construct a nomogram that could be used to calculate the OS in HCC patients. The C-index was superior to that from previous studies (0.75 vs 0.717, 0.676). Furthermore, LCAT was found to be correlated with T stage and new tumor events, and RPS6KA6 was found to be correlated with T stage.

**Conclusion:**

We identified novel therapeutic targets and constructed an individual prognostic model that can be used to guide personalized treatment in HCC patients.

## Background

Liver cancer is the sixth most common cancer and was the third major cause of cancer-related death in 2018 [1]. Hepatocellular carcinoma (HCC), the most common type of primary liver cancer, is the fourth most commonly diagnosed cancers in men and the fourth leading causes of cancer-related death among both women and men in China; the global incidence of HCC is predicted to exceed a million cases per year by 2025 [2, 3]. Alcohol abuse, hepatitis B virus or hepatitis C virus infection are the main causes of HCC. At present, surgical resection, ablative electrochemical therapies, chemoembolization, and radioembiolization are the most common treatments for HCC patients [4]. However, the 5-year survival rate of HCC patients remains poor due to intrahepatic spread and recurrence [5]. Therefore, exploring novel therapeutic targets and developing a prognosis module to guide personalized treatment are still needed.

Aberrant epigenetic changes could inappropriately inhibit or activate signaling pathways, which lead to the beginning of cancer. Epigenetic changes are considered to be a crucial step in the advancement of genetic alterations and the early stage of tumor progression [6]. DNA methylation, a covalent modification of the nucleotide cytosine at the 5′ position, is the most commonly studied epigenetic mechanism. DNA hypo- or hypermethylation could result in the occurrence of malignant tumors. DNA hypomethylation is considered to be an indication of cancer cells and affects chromosomal stability and activates oncogenes [7, 8]. DNA hypermethylation is believed to contribute to decreased gene expression and transcriptional suppression [9]. DNA methylation is an important role in tumorigenesis, and drugs that target DNA methylation are being developed based on the characteristic that DNA methylation can potentially be reversed [10].

In the present study, we initially filtered methylation-driven genes with stricter standards, which provides a compelling foundation for the study. By using univariate and multivariate Cox analyses, a prognostic module was constructed to predict the risk score of prognosis in HCC patients, then internal and external validation were performed to assess the prognostic model. Next, we conducted univariate and multivariate Cox analyses to identify independent predictors from clinical factors, including risk score, in HCC patients. Moreover, we included more comprehensive clinical information compared to that provided in previous studies and utilized the least absolute shrinkage and selection operator (LASSO) algorithm to build a nomogram that could be used to predict an individual’s OS. To our knowledge, there are no previous studies exploring the relationship between the OS of HCC patients and these clinical parameters. The C-index, ROC curve and calibrate plot were used to validate that the nomogram is superior to that from previous studies. Moreover, Student’s t-test was conducted to analyze the correlation between the core methylation-driven genes and clinical parameters in HCC patients.

## Methods

### Patients and clinical data collection

In the present study, mRNA sequencing and DNA methylation data of HCC patients were obtained from The Cancer Genome Atlas (TCGA; https://portal.gdc.cancer.gov/) database. RNA sequencing data included 374 HCC samples and 50 normal liver samples. DNA methylation data (Illumina Human Methylation 450 k) included 380 HCC samples and 50 normal liver samples. In addition, we downloaded the clinical information of HCC patients (*n* = 377) from the TCGA database.

### Identification of differentially methylated and expressed genes

The limma package was used to screen the differentially methylated genes, and the edgeR package was used to identify the differentially expressed genes [11, 12]. To improve the accuracy, the differential methylation level between HCC samples and normal liver samples (△β) greater than 0.1, false discovery rate (FDR) < 0.05, fold change> 1 were used as the cutoff criteria to identify the differentially methylated genes, and FDR < 0.05 and fold change > 2 were used to identify differentially expressed genes.

### Identification of methylation-driven genes

The match function was carried out to identify the hypermethylation-low expressed genes and hypomethylation-high expressed genes. To further improve the creditability, a correlation coefficient less than − 0.3 with a *P* value less than 0.05 was used to screen the methylated-driven genes [13].

### Construction of the risk score predictive model

To screen the methylation-driven genes related to OS, we matched survival data with follow-up more than 90 days and corresponding methylation-driven gene expression of 329 HCC patients. Univariate Cox proportional hazard analysis was utilized to filter methylation-driven genes related to OS (*P* < 0.05), and the core candidate genes to construct the risk score predictive model were identified with multivariable logistic regression analysis (P < 0.05). HCC patients were separated into high-risk and low-risk groups, and the K-M method was used to analyze the groups. Moreover, Receiver operating characteristic curves (ROC) for 1 year, 2 years and 3 years were performed to assess the prognostic model.

### Internal and external validation of the predictive model

The 329 HCC patients were randomly separated into training and testing groups by“caret” package for internal validation of the predictive model [14]. K-M analysis and ROC curves for 1 year, 2 years and 3 years were conducted to assess internal validation. Moreover, the prognostic risk model was further validated in the liver cancer database (LIRI-JP) from International Cancer Genome Consortium (ICGC; https://dcc.icgc.org/) database, K-M analysis and ROC curves for 1 year, 2 years and 3 years were performed to assess the external validation.

### Integrated analysis of the risk score and clinical parameters in HCC patients

To completely identify pretreatment-independent predictors in HCC patients, the inclusion criteria for clinical factors were as follows: 1) neoadjuvant treatment was not received before the operation; and 2) the follow-up time was more than 90 days. The clinical information (age, sex, race, BMI, HCC risk factors, and surgical method) and risk score of HCC patients (*n* = 243) were integrated according to the patient ID of HCC patients after excluding missing data. Then, univariate and multivariable Cox regression analyses were conducted to identify independent prognostic indicators from the 13 clinical parameters.

### The development and assessment of the nomogram

To avoid overfitting, LASSO logistic regression was used to filter key factors from clinical factors and build a nomogram that could be used to predict an individual’s OS. C-index, ROC curve and calibration plot were used to weigh the prognostic ability of the nomogram.

### Correlation analysis between clinical factors and methylation-driven genes

To further explore the correlation between the core methylation-driven genes that were selected to construct the risk score module and clinical factors that were screened by the LASSO algorithm, Student’s t-test was conducted, and *P* < 0.05 was considered statistically significant.

## Results

### Identification of methylation-driven genes

**Fig. 1:**
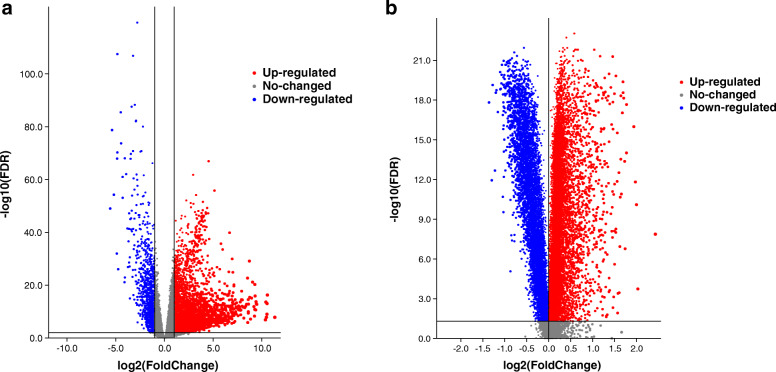
Identification of differentially expressed and methylated genes in patients with hepatocellular carcinoma. **a** The highly expressed and lowly expressed genes in hepatocellular carcinoma. **b** The hypermethylated and hypomethylated genes in hepatocellular carcinoma

**Fig. 2:**
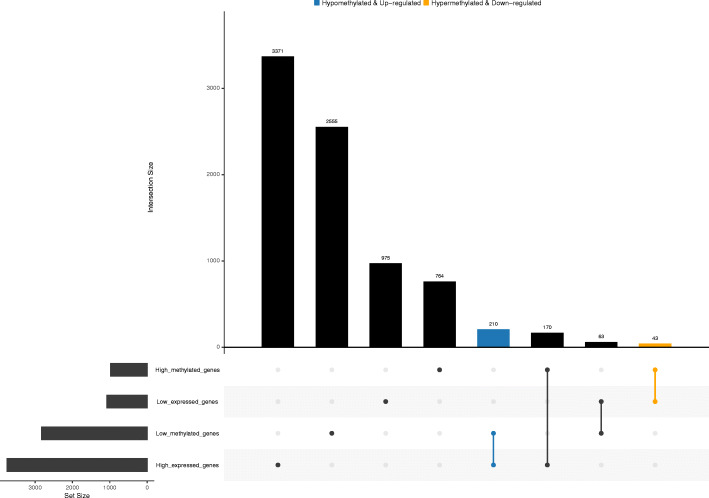
The identification of methylation-driven genes in hepatocellular carcinoma. The blue bar represents low methylated and high expressed genes. The orange bar represents high methylated and low expressed genes

**Fig. 3:**
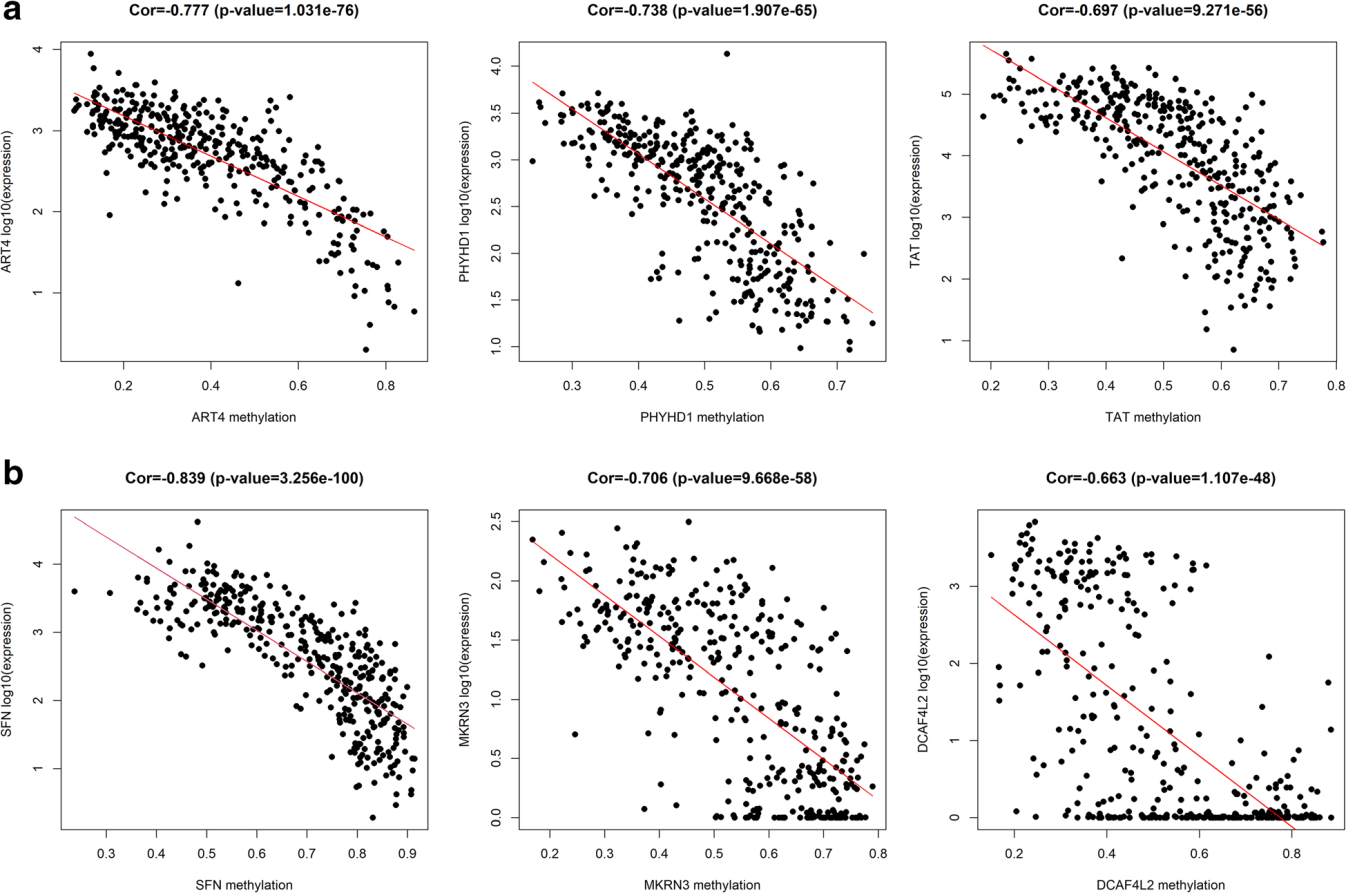
Representative methylation-driven genes in hepatocellular carcinoma. **a** Correlation between DNA methylation and genes expression of top 3 hypermethylated genes. **b** Correlation between DNA methylation and genes expression of top 3 hypomethylated genes

A total of 3751 highly expressed genes, 1081 lowly expressed genes (Fig. 1a), 977 hypermethylated genes and 2828 hypomethylated genes of HCC were extracted from the TCGA database (Fig. 1b), and 43 hypermethylation-low expressed genes and 210 hypomethylation-high expressed genes were identified (Fig. 2). Then, 44 methylation-driven genes were filtered with a correlation less than − 0.3 and *P* < 0.05, and the genes were identified to be negatively correlated with methylation level (Fig. 3).

### Construction of the risk score model

**Fig. 4:**
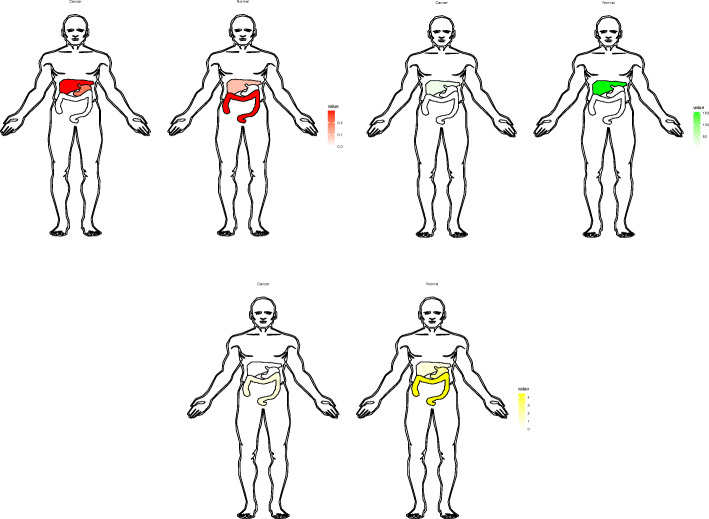
Normal represent normal liver samples from TCGA database. Cancer represent HCC samples from TCGA database. The darker the color, the higher the degree of gene expression

**Fig. 5:**
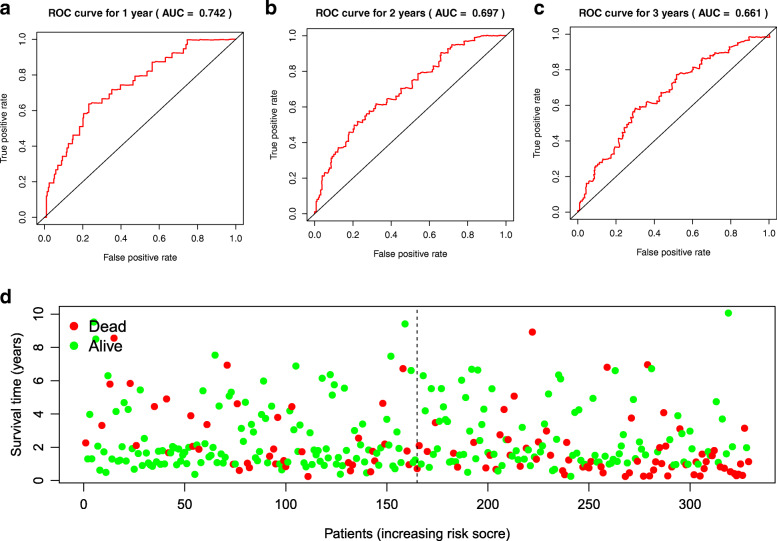
Assessment of the prognostic model in HCC patients. **a**-**c** The AUCs of the prognostic model for 1 year, 2 years and 3 years. **d** Distribution of survival status based on the prognostic model in HCC patients

**Fig. 6:**
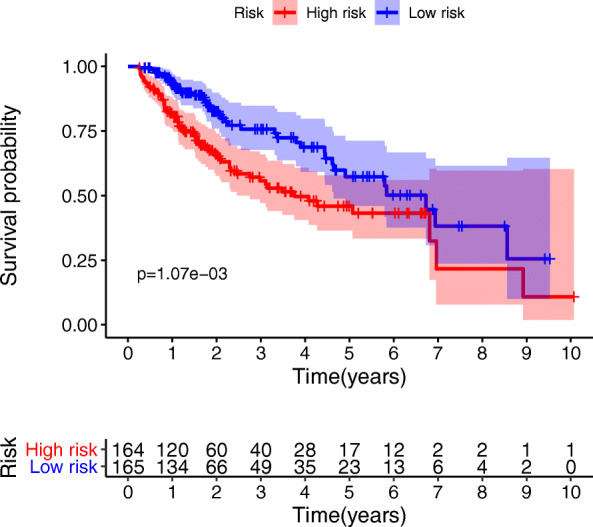
Kaplan–Meier survival analysis between high risk and low risk groups

To identify methylation-driven genes related to OS of HCC patients, survival data of 329 HCC patients and 44 methylation-driven gene expression were integrated, and C5orf58, LCAT, ADH1B, RPS6KA6, SFN and ZDBF2 were identified with univariate Cox regression analysis. The 6 methylation-driven genes were significantly related to the OS of HCC patients. To further explore the independent predictive factor, multivariable Cox regression analysis was performed based on univariate Cox analysis, and C5orf58, LCAT, and RPS6KA6 were selected (Fig. 4). Then, the risk score module was completed after the coefficients of three genes were assigned by the Cox algorithm, as follows: risk score = (0.001295 × gene level of C5orf58) + (− 0.0001 × gene level of LCAT) + (0.002257 × gene level of RPS6KA6). A total of 329 HCC patients were separated into high-risk and low-risk groups by the median risk score as the cutoff. The areas under the curves (AUC) in the prognostic model for 1 year, 2 years and 3 years were 0.742, 0.697 and 0.661, respectively (Fig. 5). The OS of the two groups was significantly different in K-M analysis (Fig. 6).

### Internal and external validation of the predictive model

**Fig. 7:**
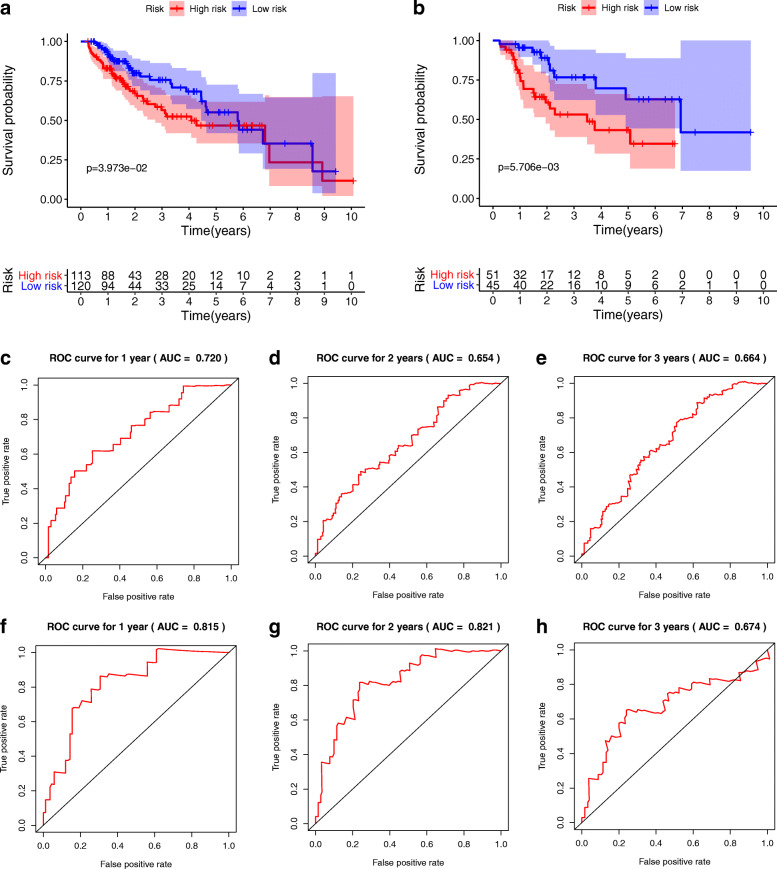
Internal validation of the prognostic model in HCC. **a **and **b** The K-M analysis of the training cohort and testing cohort. **c-e** ROC analysis of the training cohort at 1 year, 2 years and 3 years. f-h. ROC analysis of the testing cohort at 1 year, 2 years and 3 years

**Fig. 8:**
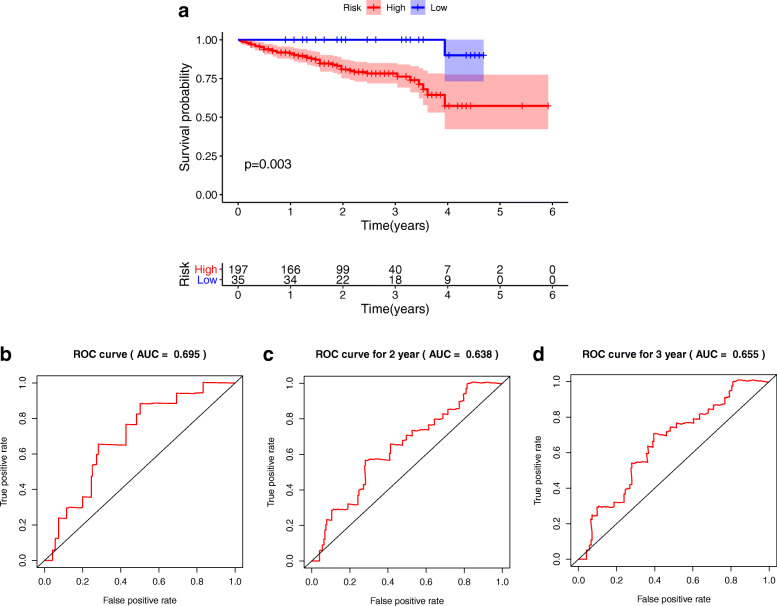
External validation of the prognostic model in HCC. **a** The K-M analysis of the external validation set. **b**-**d** ROC analysis of the external validation set at 1 year, 2 years and 3 years

The 329 HCC patients were randomly separated into training group and (*n* = 233) testing group (*n* = 96). As shown in Fig. 7a-b, the OS between high risk and low risk groups was significantly different in training and testing groups (*P* = 0.040; *P* = 0.006). Meanwhile, the AUCs in training cohort for the 1 year, 2 years and 3 years were 0.720, 0.654 and 0.664 (Fig. 7c-e), the AUCs in testing cohort for 1 year, 2 years and 3 years were 0.815, 0.821 and 0.674(Fig. 7f-h). Moreover, external validation also suggest the optimistic prognostic ability. The *P* value of K-M analysis of the high-risk and low-risk groups was 0.003 in ICGC database (Fig. 8a). The AUCs for 1 year, 2 years and 3 years were 0.695, 0.638 and 0.655, respectively (Fig. 8b-d).

### The identification of independent predictive indicators

To further assess the independence of the risk score as a predictive factor in HCC patients, risk score, age, sex, grade, T stage, BMI, race, other cancer, cancer state, HCC risk factor, surgical method, residual tumor, and new tumor events were integrated with survival information of HCC patients (*n* = 243) after excluding missing data, the characteristics of HCC patients were shown in Table 1. The 13 clinical parameters were included in univariate Cox regression analysis, and 5 clinical factors (T stage, risk score, cancer status, surgical method and new tumor events) were filtered and found to be correlated with the OS of HCC patients (Table 2). Next, four independent predictors of HCC patients (T stage, risk score, surgical method and new tumor events) were identified by the multivariable Cox regression algorithm (Table 3).

**Table 1:** Clinial characteristics of screened HCC patients

parameters	patients, n(%)
Age (years)	
< 60	163 (67.1%)
> =60	80 (32.9%)
Gender	
Female,n(%)	69 (28.4%)
Male,n(%)	174((71.6%)
Grade	
1–2,n(%)	143 (58.8%)
3–4,n(%)	100 (41.2%)
T grade	
T1–2,n(%)	182 (74.9%))
T3–4,n(%)	61 (25.1%)
Risk score	
Low,n(%)	123 (50.6%)
High,n(%)	120 (49.4%)
BMI	
< 24,n(%)	115 (47.3%)
> =24,n(%)	128 (52.7%)
Race	
Asian,n(%)	126 (51.9%)
Non-Asian,n(%)	117 (48.1%)
Other Tumor	
No,n(%)	225 (92.6%)
Yes,n(%)	18 (7.4%)
Cancer status	
Tumor Free,n(%)	162 (66.7%)
With Tumor,n(%)	81 (33.4%)
HCC risk factor	
No,n(%)	55 (22.6%)
Yes,n(%)	188 (77.4%)
Surgical method	
Lobectomy,n(%)	94 (38.7%)
Non-Lobectomy,n(%)	149 (61.3%)
Residual tumor	
R0,n(%)	230 (94.7%)
No R0,n(%)	13 (5.3%)
New tumor event	
No,n(%)	105 (43.2%)
Yes,n(%)	138 (56.8%)

**Table 2:** Univariate COX regression analyses of clinicopathologic factors associated with OS

Parameters	univariate analysis HR(95% CI)	*P* value
T stage	2.845 (1.768–4.580)	< 0.001
New tumor events	5.222 (2.584–10.555)	< 0.001
Cancer status	2.460 (1.519–3.982)	< 0.001
BMI	0.680 (0.424–1.090)	0.109
Risk score	1.814 (1.123–2.930)	0.015
HCC risk factors	0.737 (0.437–1.243)	0.252
Surgical method	0.409 (0.251–0.667)	< 0.001
Residual tumor	1.415 (0.513–3.899)	0.502
Race	1.175 (0.726–1.903)	0.512
Grade	1.130 (0.670–1.825)	0.617
Gender	0.887 (0.538–1.462)	0.639
Other tumor	1.150 (0.496–2.668)	0.744
Age	0.933 (0.567–1.536)	0.785

**Table 3:** Multivariate COX regression analyses of clinicopathologic factors associated with OS

Parameters	Multivariate analysis HR(95% CI)	*P* value
T stage	2.316 (1.352–3.968)	0.002
New tumor events	3.437 (1.476–8.001)	0.004
Risk score	1.948 (1.151–3.296)	0.013
Surgical method	0.519 (0.302–0.890)	0.017

### The development and assessment of the nomogram

**Fig. 9:**
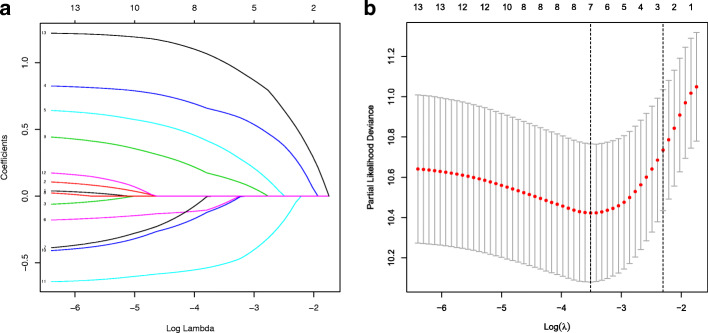
**a** Distribution of least absolute shrinkage and selection operator (LASSO) coefficients for 13 clinical parameters. **b** Partial likelihood deviation of the LASSO coefficient distribution

**Fig. 10:**
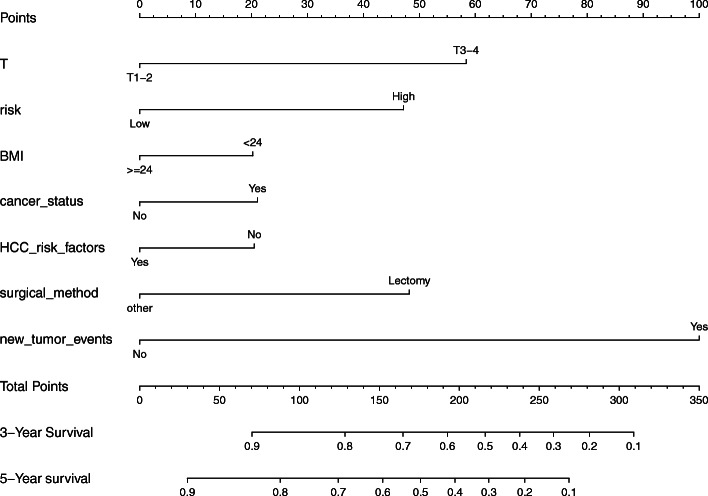
Nomogram predicting 3- and 5-year OS rates for HCC patients

**Fig. 11:**
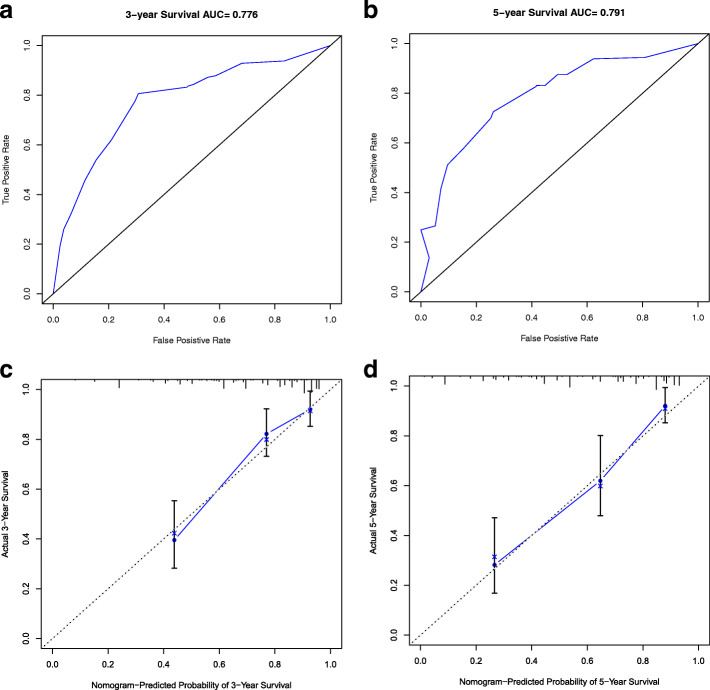
**a** and **b**. 3- and 5-year ROC curves of the nomogram. **c** and **d**. 3- and 5-y survival calibration curves of the nomogram

To determine the effect of the clinical factors for HCC patients, 13 clinical characteristics were analyzed with the LASSO logistic regression algorithm. Seven clinical factors (T stage, risk, BMI, cancer status, HCC risk factors, surgical method and new tumor events) were filtered (Fig. 9), from which a nomogram was built to predict an individual’s 3- and 5-year OS rates (Fig. 10). The reliability of the model was demonstrated by AUC (0.776, 0.791), C-index (0.75) and calibration plot (Fig. 11).

### Correlation analysis between clinical factors and methylation-driven genes

**Fig. 12:**
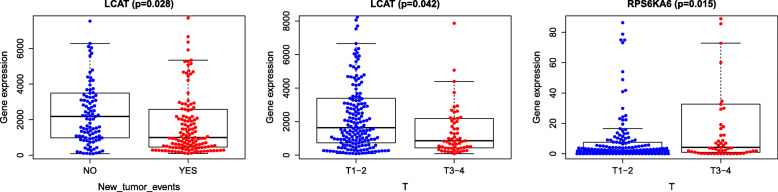
correlation between clinical parameters and core methylation-driven genes. **a** correlation between LCAT and new tumor event. **b** correlation between LCAT and T stage. c. correlation between RPS6KA6 and T stage

For further analysis of the correlation between the clinical factors that were used to construct the nomogram and the selected methylation-driven genes, Student’s t-test was performed. RPS6KA6 and LCAT were found to be correlated with T (*P* = 0.015; *P* = 0.042), and LCAT was significantly related to new tumor events (*P* = 0.028) (Fig. 12).

## Discussion

Currently, the most common treatment for HCC patients is surgical operation. However, approximately half of HCC patients with hepatectomy experience recurrence within 3 years, even at stage A [15–18]. Furthermore, metastasis before diagnosis and incomplete resection for HCC patients further reduce the 5-year survival rate. To facilitate the identification of HCC patients with poor prognosis and timely intervention implementation, the identification of predictors related to OS is urgent.

Based on the characteristic of the reversibility of DNA methylation, a large number of drugs for DNA methylation and demethylation are currently being researched. Azacitidine (AZA), a hypomethylating agent, has been suggested to be beneficial to myelodysplastic syndromes (MDS) and acute myeloid leukemia (AML) patients [19–21], and the drug is licensed for clinical treatment in the US, Europe and China. Our study used stricter criteria to identify methylation-driven genes and integrated more comprehensive clinical information, including BMI, new cancer events, surgical method, cancer state, and HCC risk factors, to predict an individual’s prognosis, which further improved the reliability of the study.

In this study, we tried to explore the biological targets of HCC that can be applied by methylation-targeted drugs. After differentially expressed genes and differentially methylated genes were identified, we found more hypomethylated genes than hypermethylated genes (2828 vs 977) and more highly expressed genes than lowly expressed genes (1821 vs 1493). Interestingly, the numbers of hypomethylated genes and highly expressed genes were greater than those in the control group, which is roughly consistent with the fact that methylation is negatively correlated with gene expression. Univariate and multivariable Cox regression analyses filtered three independent predictive genes. The high-risk group and low-risk group were separated into HCC patients using the three central methylation-driven genes. In this study, LCAT was expressed at low levels and hypermethylated in HCC samples. Lecithin-cholesterol acyltransferase (LCAT) is an important gene that is correlated with poor prognosis in many cancers, such as ovarian cancer [22], Hodgkin lymphoma [23], and breast cancer [24]. LCAT is related to fatty metabolism in males, and its activity is reduced in patients with liver disease [25]. Ribosomal protein S6 kinase A6 (RPS6KA6) is a protein in the 90-kDa ribosomal protein S6 kinase (RSK) family that participates in a series of cellular biological processes, such as cellular survival, proliferation, differentiation, mobility, nuclear signaling and protein synthesis [26–28]. RPS6KA6 plays a differential role in cancers and was reported to be an oncogene in lung squamous cell carcinoma and renal cell carcinoma [29, 30]. In contrast, RPS6KA6 works as a tumor suppressor in endometrial cancer, acute myeloid leukemia, ovarian cancer, and breast cancer [31–34]. Furthermore, the low expression of RPS6KA6 was the result of DNA hypermethylation in endometrial cancers [31]. However, the role of RPS6KA6 in HCC has not been reported in previous studies. In our study, RPS6KA6, is a hypermethylated and lowly expressed gene, was identified as a core methylation-driven gene correlated with prognosis in HCC patients. Consistent with RPS6KA6, chromosome 5 open reading frame 58 (C5orf58) was hypermethylated and expressed at low levels in HCC. However, the roles of C5orf58 have not been previously reported. C5orf58 maps on chromosome 5 at 5q35.1 according to Entrez Gene [35]. The roles of these three genes, especially RPS6KA6 and C5orf58, have not been sufficiently elucidated in HCC. Given the potentially reversible characteristic of DNA methylation, we filtered the three core methylation-driven genes to identify high-risk HCC patients, which might be therapeutic targets of epigenetic drugs and reduce the risk score of HCC patients.

There are several comprehensive studies related to methylation-driven genes in HCC. However, few studies have integrated clinical parameters and core methylation-driven genes in HCC. The advantage of the study was that stricter criteria were used to obtain more precise methylation-driven genes. Then, the prognosis module based on three genes was built, and the number of genes to build the module increased the feasibility and reduced the clinical cost. The expression, methylation and clinical data were retrieve from matched HCC patients, the feature strengthens the persuasiveness of the prognostic module based on the methylation-driven genes in HCC patients. The internal and external validation of the prognostic module also shown optimistic predictive capacities. Next, we first included more comprehensive clinical information than that included in previous studies to construct a nomogram and calculate an individual’s prognosis. The nomogram to predict prognosis in HCC patients was more accurate than that in a previous study (C-index: 0.75 vs 0.717, 0.676) [36, 37]. Furthermore, T stage, new tumor event, surgical method and risk score were filtered to be the independent prognosticators, which further strengthens the result that risk score might be utilized to calculate the prognosis in HCC patients. Finally, LCAT was found to be correlated with T stage and new tumor events, and RPS6KA6 was found to be related to T stage. It is worth further exploring whether the T stage and new cancer events were reversed by regulating LCAT and RPS6KA6. Overall, the nomogram, independent prognosticators and potential treatment targets are beneficial for individualized treatment of HCC patients.

Certainly, the potential limitations of the study should be noted. The biological mechanisms of LCAT, RPS6KA6 and C5orf58 remain to be explored. In addition, the study was based only on research data from the TCGA database, which might contribute to selection bias. Therefore, a multicenter and large-scale study should be implemented to further validate our model.

## Conclusions

In summary, a risk score module based on three core methylation-driven genes was developed, and it might be an independent predictor in HCC patients. In addition, we first included more comprehensive clinical information to construct the nomogram, which might be beneficial for predicting an individual’s OS. Moreover, the independent predictors were identified from clinical parameters. Finally, core methylation-driven genes related to clinical parameters were demonstrated.

To the best of our knowledge, a risk score module and nomogram have been proposed for the first time, and it might be beneficial to explore potential therapeutic targets and develop individualized treatments for HCC patients.

## Data Availability

The datasets used and analysed for this study were obtained from TCGA (https://portal.gdc.cancer.gov/) and ICGC (https://dcc.icgc.org/).

## Abbreviations

HCC: Hepatocellular carcinoma
TCGA: The Cancer Genome Atlas
LASSO: Least absolute shrinkage and selection operator
FDR: False discovery rate
MDS: Myelodysplastic syndromes
AML: Acute myeloid leukemia
ROC: Receiver operating characteristic curve
AUC: Areas under the curve
